# Clinical Presentation and Causes of Non-traumatic Spinal Cord Injury: An Observational Study in Emergency Patients

**DOI:** 10.3389/fneur.2021.701927

**Published:** 2021-08-09

**Authors:** Leonie Müller-Jensen, Christoph Johannes Ploner, Daniel Kroneberg, Wolf Ulrich Schmidt

**Affiliations:** ^1^Department of Neurology, Berlin Institute of Health, Charité–Universitätsmedizin Berlin, Corporate Member of Freie Universität Berlin, Humboldt-Universität zu Berlin, Berlin, Germany; ^2^Center for Stroke Research, Charité–Universitätsmedizin Berlin, Corporate Member of Freie Universität Berlin and Humboldt-Universität zu Berlin, Berlin, Germany

**Keywords:** non-traumatic spinal cord injury, myelopathy, spinal metastases, spinal lesions, multiple sclerosis, myelopathy mimic

## Abstract

**Introduction:** Diagnosing non-traumatic spinal cord injury (NTSCI) is often challenging. However, clear discrimination from non-spinal pathologies, e.g., “myelopathy-mimics” (MMs), is critical in preventing long-term disability and death. In this retrospective study we (1) investigated causes of NTSCI, (2) identified clinical markers associated with NTSCI and (3) discuss implications for NTSCI management.

**Methods:** Our sample consisted of 5.913 consecutive neurological and neurosurgical patients who were treated in our emergency department during a one-year period. Patients with a new or worsened bilateral sensorimotor deficit were defined as possible NTSCI. We then compared clinical and imaging findings and allocated patients into NTSCIs and MMs.

**Results:** Of ninety-three included cases, thirty-six (38.7%) were diagnosed with NTSCI. Fifty-two patients (55.9%) were classified as MMs. In five patients (5.4%) the underlying pathology remained unclear. Predominant causes of NTSCI were spinal metastases (33.3%), inflammatory disorders (22.2%) and degenerative pathologies (19.4%). 58.6% of NTSCI patients required emergency treatment. Presence of a sensory level (*p* = <0.001) and sphincter dysfunction (*p* = 0.02) were the only significant discriminators between NTSCI and MMs.

**Conclusion:** In our study, one-third of patients presenting with a new bilateral sensorimotor deficit had NTSCI. Of these, the majority required emergency treatment. Since there is a significant clinical overlap with non-spinal disorders, a standardized diagnostic work-up including routine spinal MRI is recommended for NTSCI management, rather than an approach that is mainly based on clinical findings.

## Introduction

Non-traumatic spinal cord injury (NTSCI) is a neurological emergency associated with a high risk for morbidity and reduced quality of life (1, 2). It is defined as any damage to the spinal cord resulting from a non-traumatic cause (3). Etiologies include degenerative, inflammatory, neoplastic and infectious conditions (1, 4–6).

**Figure 1:**
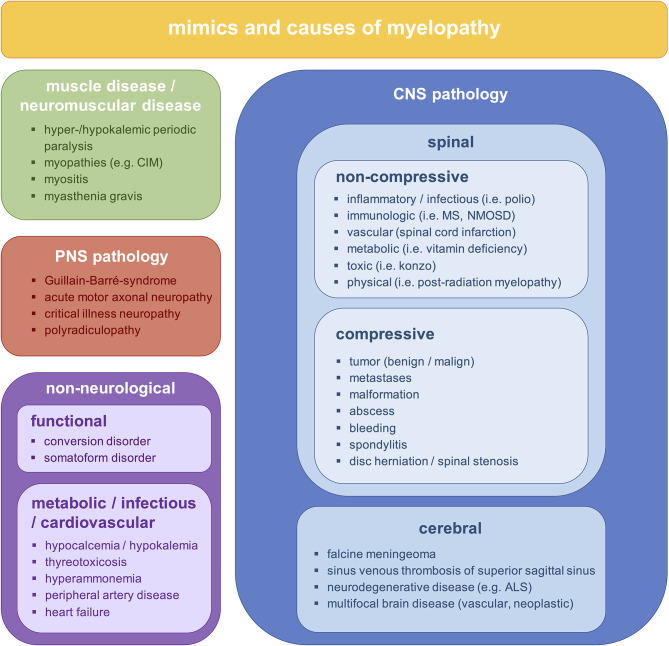
Differential diagnoses of bilateral sensorimotor deficits. ALS, amyotrophic lateral sclerosis; CIM, critical illness myopathy; CNS, central nervous system; MS, multiple sclerosis; NMOSD, neuromyelitis optica spectrum disorder; PNS, peripheral nervous system.

To prevent long-term disability and death, early diagnosis and treatment of patients with NTSCI is critical. However, diagnosing NTSCI in an emergency setting is challenging due to late consultation, oligosymptomatic presentation and a broad spectrum of non-spinal pathologies that can resemble NTSCI (myelopathy mimics, Figure 1) (7). In fact, a significant delay in diagnosing NTSCI has been reported to result from initial misdiagnosis (8–10). Since the clinical hallmark of NTSCI, a sensorimotor paraplegia with sphincter dysfunction, is often missing, critical conditions like spinal hematomas or spinal dural arteriovenous fistulas may be mistaken for peripheral neuropathies (11–14). Furthermore, psychiatric diseases and metabolic disturbances may present with symptoms similar to NTSCI (15, 16). In these cases, timely and adequate treatment depends on the physician's ability to differentiate reliably between NTSCI and myelopathy mimics (MM).

Even though NTSCI accounts for up to 79% of spinal cord injuries (17, 18), only limited data on underlying pathologies, presentation and differential diagnosis is available. Since functional outcome of NTSCI is generally worse compared to traumatic spinal cord injuries, standardized procedures for the emergency management of NTSCI are urgently needed (5, 19–21).

In this retrospective observational study, we investigate a cohort of consecutive patients with possible NTSCI who were admitted to our emergency department within a 12-month period. We aim to (1) investigate presentation and underlying pathologies of NTSCI, (2) identify clinical markers that may discriminate between NTSCI and myelopathy mimics and (3) derive a diagnostic pathway for the emergency management of NTSCI.

## Methods

### Patients

Our study cohort included all neurological and neurosurgical patients that were treated in the emergency department of the Campus Virchow Klinikum, Charité Universitätsmedizin Berlin, between January 1st, 2020 and December 31st, 2020.

### Inclusion Criteria

Patients were selected from the study cohort and considered possible NTSCI cases if they met the following criteria [previously described by Schwenkreis et al. (22)]:

1. symptom onset within 4 weeks or acute deterioration of previous symptoms.
2. sensory deficits of both upper and/or both lower extremities and/or
3. paraparesis of both upper and/or both lower extremities.
4. exclusion of previous trauma, acute coma and patients with definite cerebral symptoms incompatible with spinal pathology (e.g., aphasia, hemianopia, neglect, facial involvement).

Cases of possible NTSCI were then assigned to one of the following subgroups: (1) NTSCI (spinal pathology), (2) MM (non-spinal pathology) and (3) unclear pathology. NTSCI was confirmed if MRI or CT imaging showed a spinal pathology or if spinal lesions were previously known from patient's history. Due to their considerable clinical and therapeutical overlap, NTSCIs included both lesions of the spinal cord and lesions of the cauda equina (23). Cases were classified as MMs if spinal imaging was normal or implicated a peripheral pathology (e.g., contrast enhancement of the cauda equina and conus medullaris in cases of Guillain-Barré syndrome) or if clinical data suggested another diagnosis.

Patients were categorized to group 3 (unclear pathology) if they were lost to follow-up (e.g., discharged with a planned outpatient consultation or referred to another hospital), or if they had both a spinal and non-spinal pathology. In the latter cases it remained unclear, which pathology was the leading cause of the bilateral deficit. Two independent investigators (LMJ, WUS) performed the allocation using all available clinical and imaging data including MRI and CT scans. Discrepancies in the primary classification between the two investigators were resolved by consensus after a detailed reevaluation and discussion of the patient data.

### Clinical Findings

For all selected patients, we extracted demographic characteristics (age, sex), admission mode, Manchester Triage System, time from onset to consultation, clinical presentation on admission and findings from the neurological examination documented in the patient records. Clinical examinations were performed by neurologists and/or neurosurgeons in all reported cases. CT-angiography was performed in all cases where an aortic dissection was a possible differential diagnosis, emergency spinal MRI was performed when NTSCI was suspected. To evaluate the diagnostic reliability of findings from medical history and physical examination, we compared NTSCIs and MMs regarding clinical features that are regularly altered in NTSCI: ability to walk, muscle strength in MRC grades 1–5 (24), sensory deficits, motor deficits, sphincter dysfunction, presence of tendon reflexes and babinski reflex, alteration in muscle tone and preexisting neurological or hemato-oncological disease. For all NTSCI cases the American Spinal Injury Association (ASIA) impairment scale was retrospectively scored (25).

### MRI

For all patients with possible NTSCI we determined whether a spinal MRI was performed. An emergency spinal MRI was defined as MRI < 24 h from the timepoint of arrival at the emergency department (timepoint of the Manchester Triage System) to begin of MRI examination (timepoint of MRI scout). Further, we documented whether the MRI findings initiated emergency treatment (e.g., spinal surgery or high-dose intravenous steroids). The standard MRI protocol included whole spine T1/T2 imaging as well as contrast-enhanced sequences and diffusion weighted imaging if indicated.

### Statistical Analysis

We performed group comparisons between the NTSCI and MM subgroup using Fisher‘s exact tests. An alpha-level of *p* < 0.05 was defined as statistically significant. To correct for multiple comparisons, *p*-values were adjusted using the false discovery rate method (26). Statistical analyses and graph illustration were done using Graph Pad Prism (Version 7).

### Ethics Approval

A votum from the Ethics Committee of Charité-Universitätsmedizin Berlin (“Neurological Symptoms in the Emergency Department,” EA2/100/18) granted the use and analysis of data acquired during clinical routine.

## Results

Of 5.913 patients screened, ninety-three patients fulfilled our inclusion criteria for possible NTSCI. In thirty-six patients (38.7% of patients with possible NTSCI or 0.6% of all neurologic/neurosurgical patients) definite NTSCI was diagnosed, while in fifty-two patients (55.9%) a spinal pathology was excluded. In five patients (5.4%), the underlying pathology was inconclusive: Three patients were discharged with a planned outpatient consultation or referred to a different hospital, one patient had clinical signs of both a degeneration of the upper as well as the lower motor neuron and one patient presented with a high-grade spinal stenosis with myelopathy as well as an acute demyelinating polyneuropathy. For the latter two it remained unclear which pathology was the leading cause of their para- or tetraparesis.

### Etiologies of NTSCI

**Figure 2:**
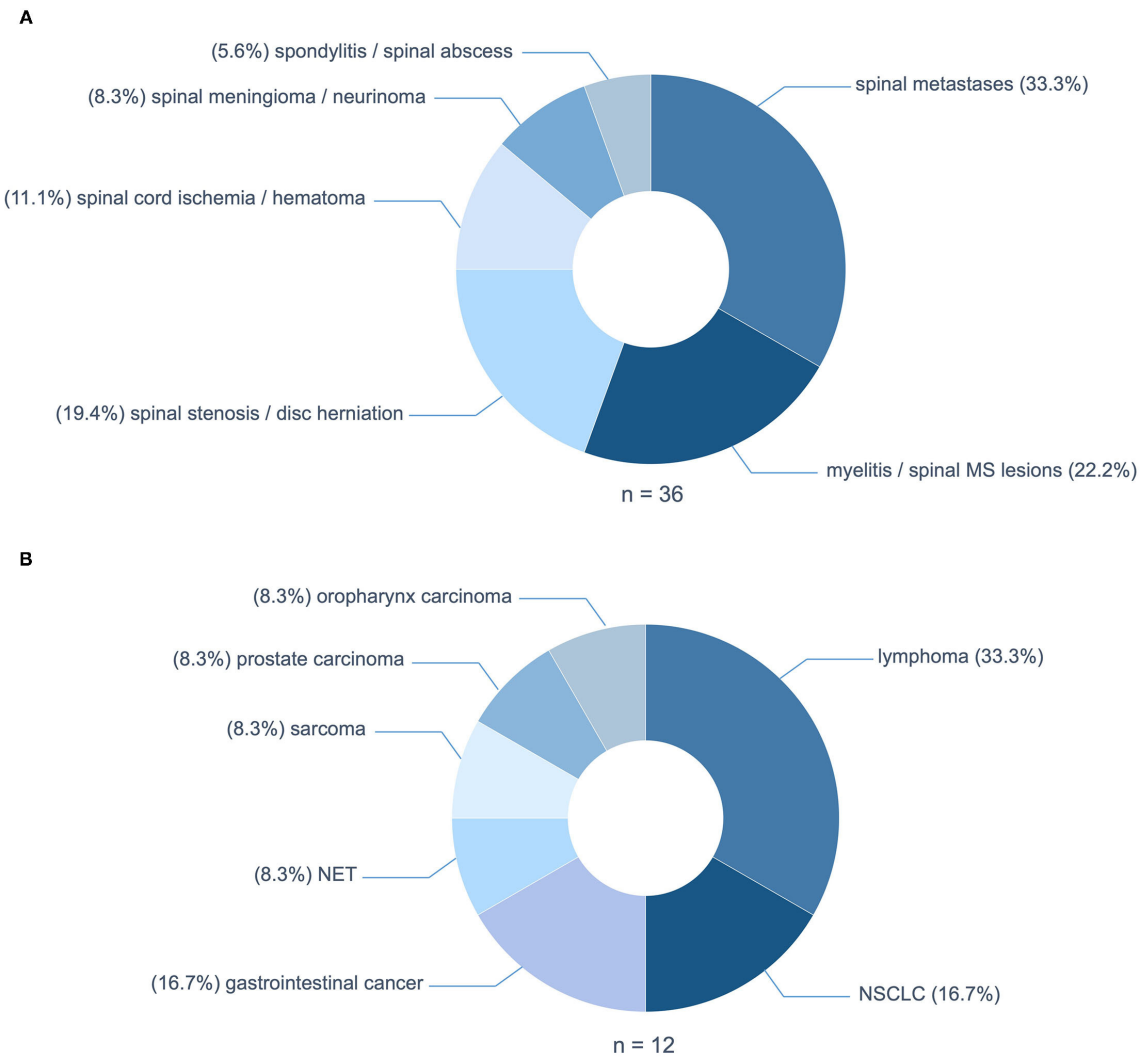
**(A)** Etiologies of non-traumatic spinal cord injury (NTSCI). **(B)** Underlying tumor entities of spinal metastases. MS, multiple sclerosis; NSCLC, non-small cell lung cancer; NET, neuroendocrine tumor.

The predominant cause of NTSCI were spinal metastases in twelve patients (33.3%) (Figure 2A). The underlying tumors were mainly lymphomas (*n* = 4; 33.3%); two cases of diffuse large B-cell lymphoma and two cases of multiple myeloma (Figure 2B). Non-small cell lung cancer and gastrointestinal cancer both accounted for 16.7% (*n* = 2) of patients with spinal tumor infiltration. Median time from cancer diagnosis to NTSCI were 31 months and the median tumor stadium at presentation was stadium IV. Intriguingly, in two patients NTSCI was the first manifestation of malignancy.

The second most frequent causes of NTSCI were inflammatory etiologies in eight patients (22.2%): five patients with spinal lesions of multiple sclerosis, one patient with transverse myelitis due to systemic lupus erythematosus (SLE), one patient with Elsberg syndrome and one patient with preexisting myelitis of unknown cause. These were followed by degenerative causes due to spinal stenosis or disc herniation with myelopathy in seven patients (19.4%; Figure 2A). Other pathologies causing NTSCI in our cohort were spinal cord ischemia and spinal hematoma in four patients (11.1%), spinal meningeoma and neurinoma in three patients (8.3%) and infectious spondylitis and spinal abcesses in two patients (5.6%; Figure 2A).

### Etiologies of Myelopathy Mimics (MMs)

**Figure 3:**
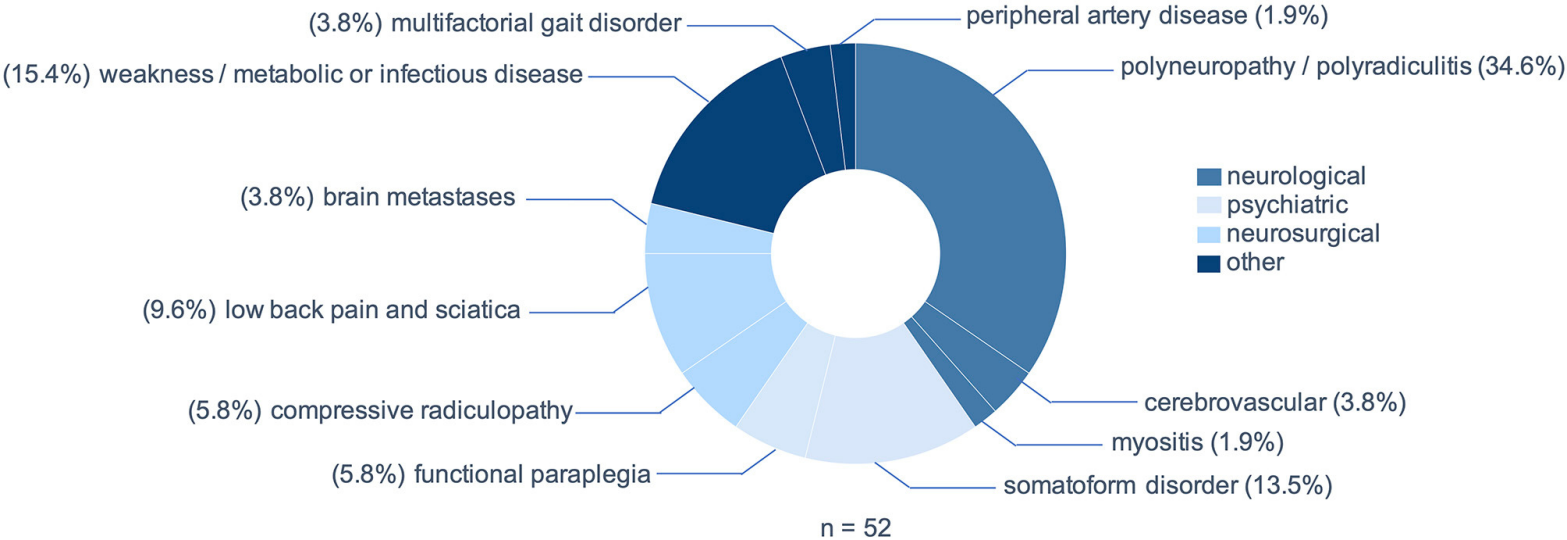
Myelopathy mimics: underlying pathologies.

Peripheral neuropathies including polyneuropathies and polyradiculitis were the most prevalent MM, including two cases of Guillain-Barré syndrome and three cases of chronic inflammatory demyelinating neuropathy (CIDP), but also acute deterioration of preexisting polyneuropathies (*n* = 18; 34.6%, Figure 3). In ten patients (19.3%) a psychiatric diagnosis was assumed as the cause of bilateral sensorimotor deficit. Of these, three patients (5.8%) presented with a sudden-onset, sensorimotor paraplegia, which was found to be of functional origin after extensive diagnostic work-up. The remaining seven patients (13.5%) presented polytopic symptoms with no evidence of cerebral, spinal or peripheral lesion and no manifestation of an alternative medical disease. Relevant differential diagnoses that usually fall within the scope of neurosurgeons were low back pain and sciatica (*n* = 5; 9.6%), compressive radiculopathies (*n* = 3; 5.8%) and cerebral metastases (*n* = 2; 3.8%). Notably, in eight patients (15.4%) generalized weakness due to metabolic alterations or infection imitated NTSCI, including one case of hyperkalemic paralysis, three cases of weakness and exsiccosis, three cases of hepatic encephalopathy and one patient with covid-19 and known multiple sclerosis, in which an Uhthoff phenomenon worsened a preexisting paraparesis.

### Clinical Data

**Figure 4:**
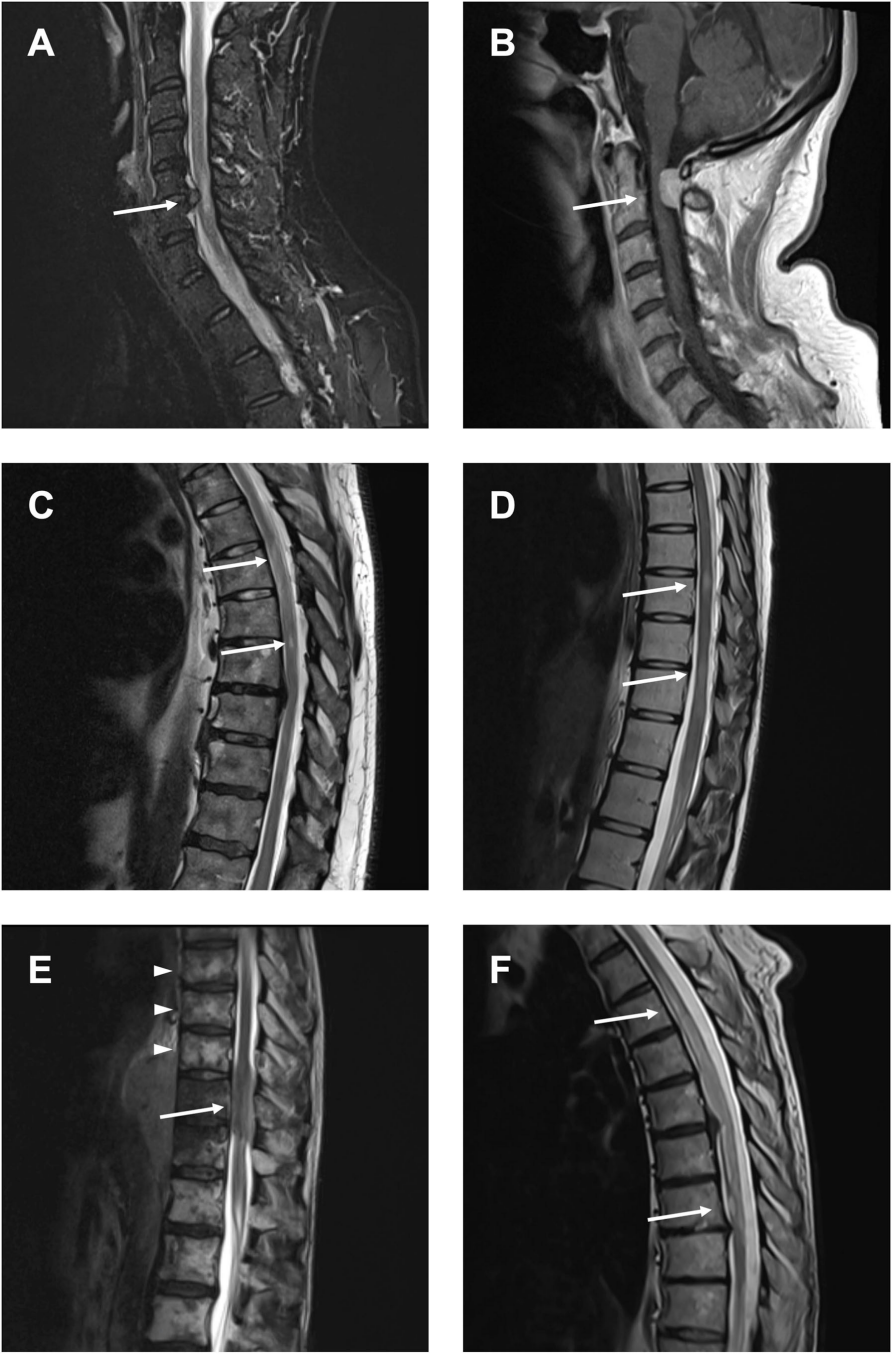
Compressive and non-compressive myelopathies causing NTSCI. **(A)** Compressive myelopathy due to cervical spinal stenosis. **(B)** Compressive myelopathy due to spinal meningioma. **(C)** Myelopathy due to spinal cord ischemia. **(D)** Spinal lesions in multiple sclerosis. **(E)** Spinal metastasis (arrow) and vertebral metastases (arrowheads) in a patient with lymphoma. **(F)** Leptomeningeal carcinomatosis in a patient with non-small cell lung cancer (NSCLC).

The two groups (NTSCI and MM) did not show significant differences regarding age, admission mode or assigned Manchester Triage System (Table 1). There were slightly more females in the NTSCI group compared to the MM group (52.8 and 38.5%, respectively). The average time from onset to clinical admission was 5.8 days (range 0–28) in both groups. Median ASIA score in the NTSCI group was D, indicating an incomplete lesion with partially preserved motor function (>50% of key muscles have a muscle grade greater or equal to MRC 3/5). Patients with definite NTSCI received spinal MRI <24 h more often than patients with MM (*p* < 0.001; Table 1). The average time to emergency MRI was not significantly different in both groups (Table 1). Exemplary MRI findings of patients with NTSCI are found in Figure 4. MRI led to acute initiation of emergency treatment (e.g., spinal surgery or intravenous steroids) in 58.6% of NTSCI patients (*n* = 17 of 29 patients with emergency MRI; Table 1). Seven patients with proven NTSCI (19.4%) did not receive emergency MRI: 3/7 patients presented with exacerbation of preexisting symptoms which could be attributed to a prior diagnosis (one case of multiple sclerosis, one case of preexisting myelitis, one case of leptomeningeal carcinomatosis), in 2/7 abdominal or spinal CT-scan revealed the pathology (one case of aortic dissection and one case of spinal tumor infiltration), one patient with preexisting multiple sclerosis and additional urinary retention as well as bilateral sensory deficit refused spinal MRI and one patient was misdiagnosed with Guillain-Barré syndrome. This 31-year-old female patient presented with lower limb paraparesis accompanied by overall reduced tendon reflexes and symmetrical sensory loss of the toes. An outpatient lumbar MRI had not shown any spinal pathology. During hospitalization, however, a whole spine contrast-enhanced MRI examination was performed and revealed a spinal meningioma with compressive myelopathy.

**Table 1:** Demographical and clinical data of patients with NTSCI and myelopathy mimics.

	**NTSCI (*n* = 36)**	**Myelopathy mimics (*n* = 52)**
Mean age in years (range)	52 (18–83)	55 (18–91)
Sex in % female	52.8%	38.5%
Mean time from onset to presentation in days (range)	5.8 (0–28)	5.8 (0–28)
Median Manchester Triage System (range)	3 (2–5)	3 (2–5)
Admission via ambulance	44.4%	55.8%
Emergency MRI	80.6%	28.8%
Mean time to emergency MRI (range)	338 (40–1,150)	350 (45–1,240)
Imaging in total (CT or MRI)	94.4%	44.2%
Emergency treatment after MRI	58.6%	n.a.
Median ASIA impairment scale score (range)	D (C-D)	n.a.

*ASIA, American spinal injury association; MRI, magnetic resonance imaging; n.a., not applicable*.

### Medical History and Neurological Examination

**Figure 5:**
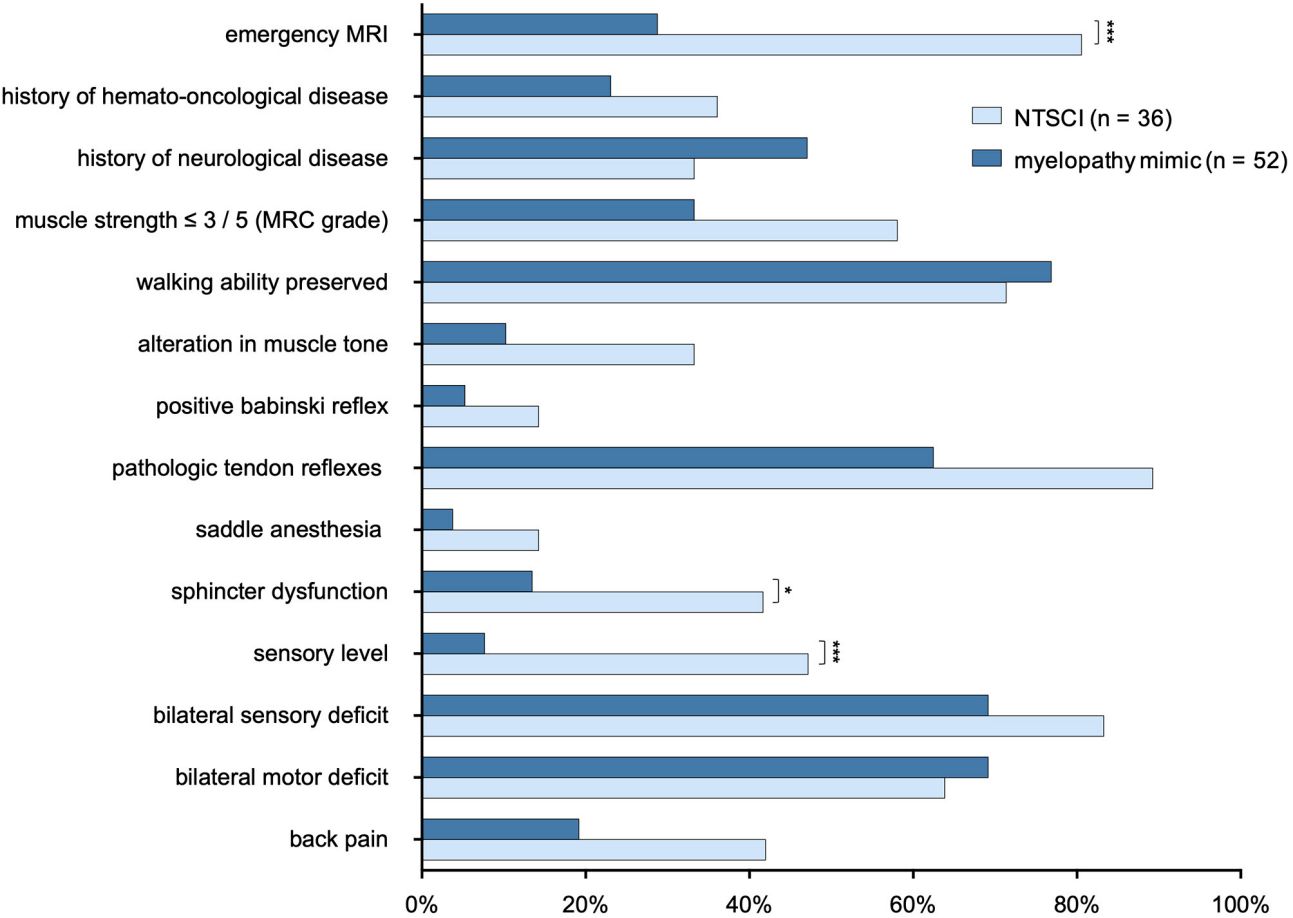
Comparison of neurological examination and medical history in patients with NTSCI and myelopathy mimics. MRC, Modified Medical Research Council; MRI, magnetic resonance imaging; *p < 0.05; ***p <0.001 (*p*-values adjusted for multiple comparisons using false discovery rate (Benjamini & Hochberg).

We compared features of the neurological status and medical history between patients with NTSCI and MMs. Presence of a sensory level and sphincter dysfunction were significantly associated with NTSCI (*p* = < 0.001 and *p* = 0.02, respectively; Figure 5). While test sensitivity was only 47.2% for presence of a sensory level and 41.7% for sphincter dysfunction, test specificity was high with 92.3 and 86.5%, respectively. Frequency of muscle strength lower than 3/5 (MRC grade), pathological tendon reflexes (this included reduced and enhanced tendon reflexes as well as asymmetrical reflexes) and back pain did not statistically differ between both groups (*p* = 0.09, *p* = 0.07, and *p* = 0.08, respectively), but showed a trend to be more prevalent in NTSCI.

### Analysis of Patients With Multiple Sclerosis

**Figure 6:**
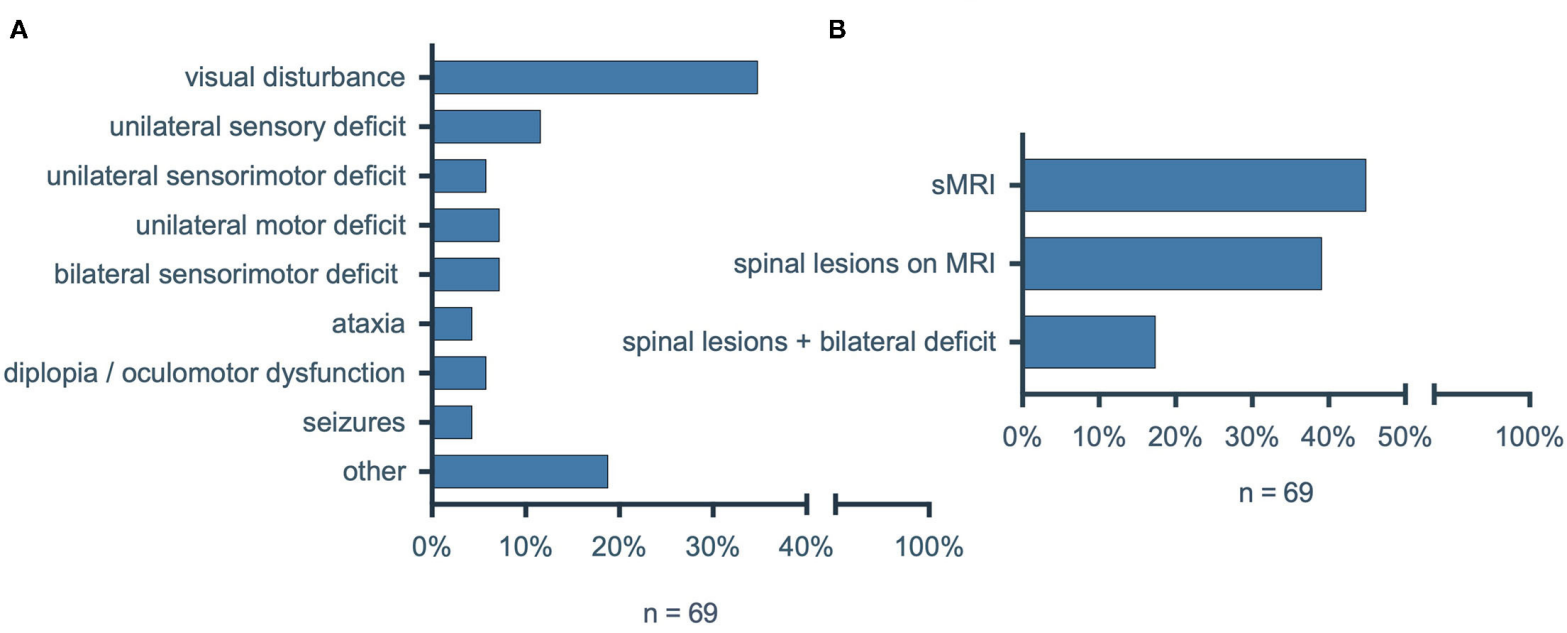
**(A)** Leading symptoms of patients with multiple sclerosis in the emergency department. **(B)** Percentage of MS patients who received spinal MRI during hospitalization, with detected spinal lesions on MRI and with diagnosed spinal lesions on MRI and a new or preexisting bilateral sensorimotor deficit. MS, multiple sclerosis; sMRI, spinal magnetic resonance imaging.

Given the high frequency of spinal involvement in multiple sclerosis (MS), we were surprised by the relatively low number of MS patients with NTSCI. We therefore conducted an additional analysis to investigate the incidence of oligosymptomatic spinal lesions in MS as an exemplary entity with a high risk for NTSCI. We included all consecutive patients with diagnosed MS that were admitted to our emergency department between January 1st, 2020 and December 31st, 2020. Of ninety-four identified patients, twenty-five patients were excluded because they presented non-neurological symptoms in the emergency department (e.g., pneumonia, cut wound). Of the sixty-nine remaining patients we collected clinical data (leading symptom, neurological status) as well as MRI findings (Figure 6A). The most prevalent symptom was visual disturbance (*n* = 24; 34.8%), while a new bilateral sensorimotor deficit was the leading symptom in only five patients (7.2%). Intriguingly, 31/69 (44.9%) of patients with MS received a spinal MRI during hospitalization and of these 27/31 (87.1%) showed spinal MS lesions (Figure 6B). Since only twelve (44.4%) of the twenty-seven patients with diagnosed spinal MS lesions on MRI had any bilateral sensorimotor deficit (new, preexisting or in the past), fifteen (55.6%) had oligosymptomatic spinal lesions with no or only unilateral sensorimotor deficits.

## Discussion

In this retrospective, single-center observational study, we diagnosed non-traumatic spinal cord injury (NTSCI) in 0.6% of all neurological or neurosurgical cases that presented in our emergency department within a period of 12 months. Given this percentage, there are at least two cases of NTSCI every month in a hospital with 5,000–6,000 neurological and neurosurgical patients per year.

The three most frequent underlying pathologies in our sample were (1) spinal metastases (33.3%), (2) inflammatory spinal cord lesions (22.2%) and (3) compressive myelopathy due to spinal stenosis or disc herniation (19.4%). Similar proportions have been reported by New et al. in their sample of 134 NTSCI patients: 20.1% had a spinal tumor manifestation, 19.4% had NTSCI caused by MS and in 17.9% a degenerative pathology was diagnosed (6). In other studies, degenerative spine disease has been reported as the predominant cause of NTSCI, ranging from 28.3% in a study by Buzzell et al. to 54.0% in a study by McKinley et al. (1, 6, 18). Inflammatory conditions made up 2.0–23.0% and spinal metastases 16.4–26.0% of NTSCI cases (1, 6, 18, 27). Interestingly, a recent study showed that in patients with NTSCI that required intensive care unit treatment, infectious and inflammatory causes were most common (2).

However, most of the studies were published more than 10 years ago, at a time when MR imaging was frequently not available. Hence, it can be hypothesized that demographic changes and the higher prevalence of patients with extensive tumor disease due to new target therapies have led to an increase of spinal metastases causing NTSCI. At the same time, improved spinal imaging might have resulted in earlier diagnosis and surgical treatment of non-compressive spinal stenosis, therefore preventing NTSCI caused by degenerative pathologies.

Our inclusion criteria for possible NTSCI required a bilateral sensory and/or motor deficit. However, the additional analysis of MS patients showed that 55.6% of spinal MS lesions were not associated with a bilateral sensorimotor deficit. Thus, as described previously (28–31), a great number of spinal lesions remain oligosymptomatic and do not present with the hallmark of NTSCI, a bilateral deficit.

At the same time, hospitals cannot provide 24-h spinal MR imaging for all patients in the emergency department. Therefore, criteria to diagnose NTSCI with high sensitivity and specificity based on clinical features are desirable. However, our results show that many clinical findings are of limited diagnostic value. Among the investigated signs, the presence of a sensory level and sphincter dysfunction were the only parameters with high specificity for NTSCI (92.3 and 86.5%, respectively). Still, sensitivity was relatively low with 47.2% for presence of a sensory level and 41.7% for presence of sphincter dysfunction. Other parameters such as back pain and preexisting hemato-oncological disease can be considered “red flags” for NTSCI; however, they did not reach statistical significance in our analysis.

Since significantly more MRIs were performed in the NTSCI group compared to the MM group, we have to assume that additional clinical factors exist that were implicitly used by physicians in the emergency department but were not analyzed in this study. One possible distinctive feature might be symptom severity. Our data did not reveal relevant differences in the preservation of walking ability; however, the percentage of patients with a high-grade motor deficit (MRC muscle grade ≤ 3/5) tended to be more common in patients with NTSCI. In the literature, multiple scoring systems for the severity of spinal cord injuries exist (25, 32). Still, the widely used ASIA impairment scale and the Walking index for spinal cord injury (WISCI) focus on motor deficits and are designed for the assessment of traumatic spinal cord injuries. This was also reflected in the ASIA scoring of our NTSCI patients: There were only patients with ASIA score C and D (defined as C = “motor function is preserved below the neurological level, and more than half of the key muscles below the neurological level have a muscle grade <3” and D = “motor function is preserved below the neurological level, and at least half of key muscles below the neurological level have a muscle grade greater than or equal to 3”) (25). This demonstrates that most NTSCI patients present with an incomplete spinal cord injury. Therefore, more sensitive scores that also emphasize sensory deficits may improve clinical assessment of patients with possible NTSCI.

Our study provides an overview of the heterogenic spectrum of MMs. In the literature only case reports and review articles on differential diagnosis of NTSCI exist (7, 16, 22). In our sample, peripheral nerve disorders were the predominant MM. However, in 19.3% (*n* = 10) a psychogenic impairment was assumed. In 5.8% (*n* = 3) a functional origin of paraplegia was concluded, after extensive diagnostic work-up did not reveal a spinal or non-spinal pathology. Heruti et al. described thirty-four patients with mono-, para- or tetraplegia of which thirty had a conversion disorder while four were diagnosed as malingerers (15). In these patients, inaccurate diagnostic labeling may provoke unnecessary and potentially harmful treatments. Hence, functional impairment needs to be considered early in the diagnostic process, if clinical presentation appears implausible for a neurological syndrome.

A decisive diagnostic approach is not only vital in psychiatric differential diagnoses: In our sample, three patients with Guillain-Barré syndrome, one patient with critical hyperkalemia and one patient with acute artery occlusion of the lower extremities presented as MM. In all cases, emergency treatment was required. Consequently, when spinal MRI does not detect an explanatory spinal pathology, additional diagnostic measures (e.g., lumbar puncture, blood tests) have to be taken to rule out critical MMs (7).

In summary, approximately one-third of emergency patients presenting with a new bilateral sensorimotor deficit will have an underlying spinal pathology. Of these, the majority will need emergency treatment.

**Figure 7:**
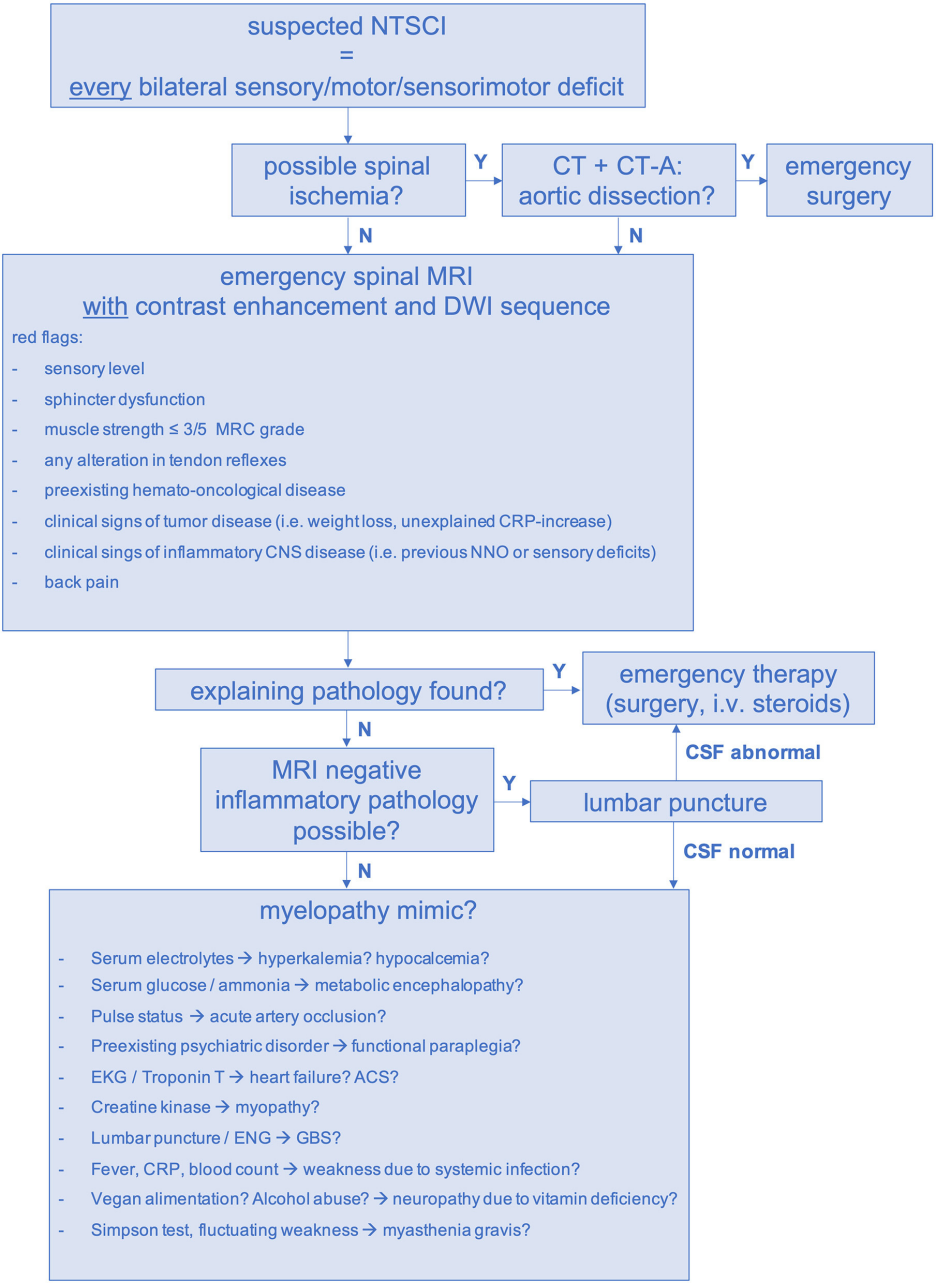
Diagnostic pathway for suspected NTSCI in the emergency setting. ACS, acute coronary syndrome, CNS, central nervous system; CRP, c-reactive protein; CT, computed tomography scan; CT-A, computed tomography angiogram; DWI, diffusion-weighted imaging; ENG, electroneurography, GBS, Guillain-Barré syndrome; MRI, magnetic resonance imaging; N, no; NNO, optic neuritis; NTSCI, non-traumatic spinal cord injury; i.v., intravenous; Y, yes.

Considering the high frequency of oligosymptomatic spinal cord lesions, the limited value of individual clinical markers and examination findings has to be acknowledged. Hence, we propose a standardized diagnostic pathway for the management of suspected NTSCI in the emergency setting (Figure 7). In our study, we had to conduct forty-four emergency MRIs in order to find twenty-nine spinal pathologies (number needed to image = 2). Of course, it has to be considered that thirty-seven patients were allocated to the MM group and did not receive an emergency MRI. Thus, the possibility of false-negative MMs remains. Considering this, performing emergency MRI in all patients with an unexplained bilateral sensory, motor or sensorimotor deficit is warranted. If available, MRI scans should be performed with contrast enhancement to facilitate detection of inflammatory causes, leptomeningeal carcinomatosis and spinal metastases (33). Additionally, DWI-MRI is necessary to rule out spinal ischemia in cases of sudden-onset paraplegia (34). When aortic dissection is possible, CT-angiography should precede spinal MRI (Figure 7) (35).

In addition, given the late consultation of NTSCI patients (mean = 5.8 days after symptom onset), we suggest that high-risk cohorts, e.g., patients with MS and malignancies, should be routinely educated on signs and symptoms of NTSCI. In addition, existing guidelines on the routine implementation of spinal MRIs in asymptomatic MS and tumor patients should be realized (36–38). In certain cases, the detection of clinically unapparent spinal lesions will enable the diagnosis of multiple sclerosis instead of clinical isolated syndrome (CIS) and thus allow an earlier initiation of a disease-modifying therapy (39, 40). In oncological patients, early diagnosis of spinal metastases significantly impacts therapeutical decisions such as continuation of tumor therapy vs. initiation of palliative care.

Our study has several limitations. First, this is a retrospective analysis without controlled study conditions and with a relatively small sample size. Further, the hospital's level of care and thus the spectrum of specialist departments (e.g., department of neurosurgery and neurology) influences the selection of patients allocated to the emergency ward. For example, a small-scale hospital without a neurosurgical department would receive fewer patients with suspected degenerative spinal myelopathy. Furthermore, some underlying pathologies vary in their geographical, epidemiological and ethnic distribution. Hence, our data may not be applicable in other countries or continents. In Africa, for instance, 25% of NTSCI are caused by spinal manifestation of tuberculosis (Pott‘s disease). Infection with HTLV (human t-lymphotropic virus (1), syphilis and schistosomiasis are much more common than in western countries (20, 41). Consequently, multi-center studies with greater sample sizes are needed to collect representative epidemiological data on NTSCI incidence and etiologies.

## Data Availability Statement

The raw data supporting the conclusions of this article will be made available by the authors, without undue reservation.

## Ethics Statement

The studies involving human participants were reviewed and approved by a votum from the Ethics Committee of Charité-Universitätsmedizin Berlin (Neurological Symptoms in the Emergency Department, EA2/100/18). Written informed consent for participation was not required for this study in accordance with the national legislation and the institutional requirements.

## Author Contributions

LM-J, CP, and WS designed the project and interpreted the data. LM-J, WS, and DK performed the data analyses. LM-J wrote the manuscript and final responsibility for decision to submit publication. CP, WS, and DK reviewed the manuscript. All authors contributed to the article and approved the submitted version.

## Conflict of Interest

The authors declare that the research was conducted in the absence of any commercial or financial relationships that could be construed as a potential conflict of interest.

## Publisher's Note

All claims expressed in this article are solely those of the authors and do not necessarily represent those of their affiliated organizations, or those of the publisher, the editors and the reviewers. Any product that may be evaluated in this article, or claim that may be made by its manufacturer, is not guaranteed or endorsed by the publisher.
